# Safety and efficacy of anti-EGFR monoclonal antibody (SCT200) as second-line therapy in advanced esophageal squamous cell carcinoma

**DOI:** 10.20892/j.issn.2095-3941.2021.0388

**Published:** 2022-01-12

**Authors:** Ming Bai, Meng Wang, Ting Deng, Yuxian Bai, Kai Zang, Zhanhui Miao, Wenlin Gai, Liangzhi Xie, Yi Ba

**Affiliations:** 1 Tianjin Medical University Cancer Institute and Hospital, National Clinical Research Center for Cancer, Tianjin’s Clinical Research Center for Cancer, Key Laboratory of Cancer Prevention and Therapy, Tianjin 300060, China; 2 Department of Gastrointestinal Oncology, Harbin Medical University Cancer Hospital, Harbin 150081, China; 3 Department of Medical Oncology, Henan Cancer Hospital, The Affiliated Cancer Hospital of Zhengzhou University, Zhengzhou 450008, China; 4 Oncology Department, The First Affiliated Hospital of Xinxiang Medical University, Xinxiang 453100, China; 5 Sinocelltech Ltd., Beijing 100176, China; 6 Beijing Engineering Research Center of Protein and Antibody, Beijing 100176, China; 7 Cell Culture Engineering Center, Chinese Academy of Medical Sciences & Peking Union Medical College, Beijing 100176, China

**Keywords:** Epidermal growth factor receptor, esophageal squamous cell carcinoma, SCT200, monoclonal antibody

## Abstract

**Objective:**

The mainstay treatment of esophageal squamous cell carcinoma (ESCC) involves chemotherapy and immunotherapy. However, alternative therapies are required for patients who are refractory or intolerant to existing therapies.

**Methods:**

In this single-arm, multicenter, open-label phase Ib study, 30 patients received an intravenous infusion of SCT200, an anti-epidermal growth factor receptor (EGFR) monoclonal antibody, 6.0 mg/kg once a week for 6 weeks, followed by 8.0 mg/kg once every 2 weeks until disease progression or intolerable toxicity. The primary endpoint was the objective response rate (ORR). The secondary endpoints were progression-free survival (PFS), overall survival (OS), and safety.

**Results:**

Thirty patients were enrolled between July 2018 and May 2019. The ORR was 16.7% (95% CI: 5.6%–34.7%). The median PFS and OS were 3.1 months (95% CI: 1.5–4.3) and 6.8 months (95% CI: 4.7–10.1), respectively. A numerical difference without any statistical significance in ORR was observed in patients with different EGFR expressions (≥ 50%: 25.0% *vs.* < 50%: 0%, *P* = 0.140) or TP53 mutation abundance (< 10%: 23.8% *vs.* ≥ 10%: 0%, *P* = 0.286). Improved median PFS (3.4 *vs.* 1.4 months, *P* = 0.006) and OS (8.0 *vs.* 4.2 months, *P* = 0.027) were associated with TP53 mutation abundance of < 10%. The most common treatment-related adverse events of grade 3 or 4 (occurring in ≥ 2 patients) were hypomagnesemia [7 (23.3%)] and rash [2 (6.7%)]. No treatment-related death occurred.

**Conclusions:**

SCT200 monotherapy as the second- or further-line treatment for advanced ESCC showed favorable efficacy, with an acceptable safety profile. TP53 mutation abundance might serve as a potential predictive biomarker.

## Introduction

Esophageal cancer ranks seventh in incidence. With 572,000 new cases and 509,000 deaths in 2018; it is the sixth leading cause of cancer mortality worldwide^1^. More than half of global new cases and esophageal cancer-related deaths have been reported in China^1,2^. Esophageal squamous cell carcinoma (ESCC) is the predominant histological type, accounting for approximately 90% of esophageal cancer cases^3^. The standard first-line treatment for unresectable locally advanced or metastatic ESCC is a combination of fluoropyrimidine and platinum^4,5^. However, disease progression cannot be avoided. Current preferred second-line therapies include chemotherapy and immunotherapy^4,5^. The survival benefits of second-line chemotherapy with docetaxel, paclitaxel, or irinotecan monotherapy are limited for advanced ESCC, with a median overall survival (OS) of 5.3–6.1 months^6,7^. The approval of immune checkpoint inhibitors (ICIs), such as camrelizumab^8^ and pembrolizumab^9^, in China, provides improved prognosis compared with chemotherapy for patients with advanced ESCC. However, a certain number of patients show no response to immunotherapy. Therefore, novel treatment strategies, in addition to ICIs, still need to be developed, especially for patients who cannot tolerate chemotherapy.

The epidermal growth factor receptor (EGFR) gene is a classical target for cancer therapy. Approximately 33%–68% of patients with ESCC overexpress the EGFR^10–13^, which is associated with poor prognosis^11–13^. Anti-EGFR monoclonal antibodies (such as cetuximab and nimotuzumab) are EGFR-targeting therapies that are often used in combination with chemotherapy and/or radiotherapy. The addition of cetuximab or nimotuzumab to chemoradiotherapy has shown promising efficacy in patients with ESCC in single-arm trials^11,14–17^; however, they lacked survival benefits compared with chemoradiotherapy alone in randomized controlled trials (RCTs) of patients with esophageal cancer^18,19^. Further studies are therefore required to assess the function of anti-EGFR monoclonal antibodies in treating ESCC.

SCT200 is a novel recombinant humanized anti-human EGFR monoclonal antibody developed by Sinocelltech Ltd. (Beijing, China). Mechanistically, SCT200 suppresses the proliferation of cancerous cells by effectively blocking ligands such as EGF and inhibiting the activation of the EGFR signal pathway^20^. SCT200 shows significantly better antibody-dependent cell mediated-cytotoxicity (ADCC) than cetuximab. Moreover, SCT200 can stimulate the immune effects of complement-dependent cytotoxicity (CDC) and ADCC to kill tumor cells through Fc functional regions, with a killing of more than 30%. Previous studies have shown that the pharmacodynamic effects of SCT200 *in vivo* and *in vitro* correlated with its mechanism of blocking the EGFR signal pathway^20^. Regarding the safety of SCT200, the toxic target organs are mainly the skin and gastrointestinal system. There was no other non-target related toxic effect, and no obvious toxic and side effects (NOAEL) of SCT200 were found in a nonclinical safety study, highlighting the adequate safety profile of SCT200. We conducted an open-label phase I trial (Registration No. NCT02211443) to evaluate the safety, tolerability, pharmacokinetics, and preliminary efficacy of single and multiple doses of SCT200 in patients with metastatic colorectal cancer refractory or intolerant to fluoropyrimidine-, oxaliplatin-, and irinotecan-based chemotherapy. Preliminary efficacy was analyzed in 37 patients, including 22 in the dose-escalation stage and 15 in the dose-expansion stage. Data from an unpublished study showed that the objective response rate (ORR) in the dose-expansion cohort was 73.3% (11/15). Safety was analyzed in 35 patients, and treatment-related adverse events (TRAEs) were found in 33 (94.3%) patients. Dose reduction or withdrawal occurred in 11 (31.4%) patients. The majority of TRAEs were grade 1 or 2. The incidence of dermal toxicity for SCT200 was comparable to that for panitumumab and cetuximab, with lower severity. We did not observe side effects such as diarrhea, dehydration, or interstitial lung disease.

Here, we evaluated the efficacy and safety of SCT200 in patients with advanced ESCC, who were refractory or intolerant to chemotherapy with platinum, taxane, or fluoropyrimidine.

## Materials and methods

### Study design and treatment

This was a single-arm, multicenter, open-label phase Ib trial (ClinicalTrials.gov Identifier: NCT03817567) in patients with advanced ESCC after the failure of chemotherapy. Patients were recruited from 4 sites (Tianjin Medical University Cancer Institute & Hospital, Harbin Medical University Cancer Hospital, The Affiliated Cancer Hospital of Zhengzhou University & Henan Cancer Hospital, and The First Affiliated Hospital of Xinxiang Medical University) in China between July 2018 and May 2019. We conducted an open-label phase I trial (Registration No. NCT02211443) to evaluate the safety, tolerability, pharmacokinetics, and preliminary efficacy of single and multiple doses of SCT200 in patients with metastatic colorectal cancer, who were refractory or intolerant to fluoropyrimidine-, oxaliplatin-, and irinotecan-based chemotherapy. The results showed that patients could tolerate 8.0 mg/kg SCT200 once every 2 weeks for 3 weeks. Pharmacokinetic results of SCT200 showed a peak valley concentration of 6 mg/kg QW for 6 weeks in the multiple administration stage, combined with a half-life study of SCT200, suggesting that 6 mg/kg SCT200 administered once a week, reached a steady-state after the fifth administration. Moreover, the steady-state trough concentrations of cetuximab and panimab were 41–85 μg/mL and 50 μg/mL, respectively^21^. Based on the these results, eligible patients received an intravenous infusion of 6.0 mg/kg SCT200 once a week for 6 weeks, followed by 8.0 mg/kg SCT200 once every 2 weeks, until disease progression or intolerable toxicity.

The study was conducted in accordance with the Declaration of Helsinki and Good Clinical Practice. It was approved by the ethics committee of each participating institute/hospital. Written informed consent was obtained from each patient.

### Patients

Patients were eligible if their age ranged from 18−75 years, if they had histologically or cytologically confirmed locally advanced or metastatic ESCC, had undergone failed standard chemotherapy with platinum, taxane, or fluoropyrimidine, or concurrent radiochemotherapy (defined as disease progression or intolerable toxicity occurring during the treatment period or within 3 months after the last treatment), had at least 1 measurable lesion according to the Response Evaluation Criteria In Solid Tumors (RECIST, version 1.1)^22^, had an Eastern Cooperative Oncology Group (ECOG) performance status of 0–1, had a life expectancy ≥ 3 months, and had adequate organ functions [neutrophil count ≥ 1.5 × 10^9^/L; platelet count ≥ 75 × 10^9^/L; hemoglobin ≥ 80 g/L; alanine transaminase and aspartate transaminase ≤ 3 × upper limit of normal (ULN) for patients without liver metastases, and ≤ 5 × ULN for patients with liver metastases, total bilirubin ≤ 1.5 × ULN, creatinine ≤ 1.5 × ULN, and magnesium ≥ lower limit of normal]. The key exclusion criteria were a current or history of central nervous system metastases; other malignancies within 5 years, except for cured non-melanoma skin cancer, cervical cancer *in situ*, and prostate intraepithelial neoplasm; previous anti-EGFR therapy; major surgery within 4 weeks before enrollment; a history of interstitial lung disease; active hepatitis B/C; human immunodeficiency virus infection; active or uncontrolled infection within 2 weeks before enrollment, except for urinary tract and upper respiratory tract infections; or pregnant or lactating women.

### Endpoints and assessment

Patients were followed-up after the completion of the first 6-week treatment and every 8 weeks thereafter. The primary endpoint was ORR, defined as the percentage of patients with complete response (CR) and partial response (PR). The secondary endpoints were disease control rate (DCR), duration of response (DOR), time to progression (TTP), progression-free survival (PFS), OS, safety, and immunogenicity. DCR was defined as the percentage of patients with CR, PR, and stable disease (SD). DOR was defined as the time from the first documented CR or PR to disease progression or any-cause death, whichever occurred first. TTP was defined as the time from the initiation of SCT200 treatment to disease progression. PFS was defined as the time from the initiation of SCT200 treatment to disease progression or any-cause death, whichever occurred first. OS was defined as the time from the initiation of SCT200 treatment to any-cause death. An exploratory analysis was used to study gene alterations.

Tumor response was assessed by the investigator, according to RECIST 1.1^22^. Adverse events from the first dose to 28 days after the last dose were recorded and graded according to the National Cancer Institute Common Terminology Criteria for Adverse Events, version 4.03. An electrochemiluminescent bridging assay was performed to detect anti-SCT200 antibodies (ADA) in human serum samples. The capture reagent working fluid [Biotinylated (Bio)-SCT200], detecting reagent working fluid [Ruthenylated (Ru)-SCT200], and neutralization liquid (Tris-HCl, pH = 9.5) were added sequentially in a polypropylene microplate. The acidified sample was then added. By mixing these solutions, ADA bound to both Ru-SCT200 and Bio-SCT200 molecules forming an antibody complex bridge, called “Bio-SCT200~ADA~Ru-SCT200,” and then the complex bound to the streptavidin-MSD plate. With the addition of MSD Read Buffer T (MSD, Rockville, MD, USA), the ruthenium label produced a chemiluminescent signal proportional to the ADA concentration. The chemiluminescent signal was then examined to measure the concentration of ADA.

### Immunohistochemistry

EGFR expression was determined using a rabbit monoclonal antibody against the EGFR (Clone 5B7; Ventana Medical Systems, Oro Valley, AZ, USA) according to the manufacturer’s instructions. Secondary antibodies were used from the NovoLink Max Polymer Detection System (RE7280-K; Leica, Wetzlar, Germany) and the UltraView Universal DAB Detection Kit (Ventana Medical Systems). The Ventana Medical Systems BenchMark ULTRA/XT was used for immunohistochemical staining. In this study, the percentage of positive staining cells was determined. In brief, a dark brown color indicated positive staining, while negative staining was assumed when fine granular, scant, or no background staining appeared. Taking the nuclear area for analysis because the EGFR in the membranes were stained, the average value of the 5 images was used for the ratio of positive cells.

### Next-generation sequencing (NGS)

A customized 45 gene panel (Amoy Diagnostics, Shanghai, China) was used for NGS. The cell-free DNA (cfDNA) was extracted from whole peripheral blood and collected in a 10 mL cfDNA protection tube (Amoy Diagnostics). The NGS was performed using the following: the plasma was separated from the samples, and cfDNA was isolated from the plasma using the QIAamp Circulating Nucleic Acid Kit (Qiagen, Hilden, Germany). Next, a library was prepared using the NEBNext UltraII DNA library Prep Kit (Illumina, San Diego, CA, USA), and the target region was enriched with probes of the 45-gene panel, followed by sequencing with Illumina Nextseq500. Then, bioinformatics analyses based on quality control and alignment, assessment of off-target reads, marking and removal of polymerase chain reaction duplicates, realignment base score recalibrations, and estimation of sequencing coverages were performed to determine the quality of the sequencing data and variants of samples using bioinformatics workflow ADX45Gene_v0.2.0. Variants were evaluated at a frequency of ≥ 1 and support reads of ≥ 2. The entire procedure was performed at the Amoy Diagnostics Medical Institute (Shanghai, China).

### Statistical analysis

This study was a single-arm phase Ib trial, and only 30 cases were included in the study. Moreover, the number of patients enrolled in stage Ib included only 30 cases. Considering the possible decrease in the number of cases, no statistical assumptions were made due to the difficulty of achieving a sufficiently significant level (α) with sufficient power (1-β). The sample size could not be calculated due to the exploratory nature of the study. Efficacy was analyzed using a full analysis set (FAS), which included all patients with at least 1 dose of the study drug. The safety set included all patients with at least 1 dose of the study drug and at least 1 safety assessment. Continuous variables are expressed as the median (range). Categorical variables are expressed as the frequency (percentage). The 95% confidence intervals (CIs) of categorical variables were estimated using the Clopper-Pearson’s method. Survival curves were plotted using the Kaplan-Meier method, and the 95% CI of survival was estimated using the Brookmeyer-Crowley method. Comparisons of ORR and survival in the subgroups by EGFR expression and TP53 mutation abundance were conducted using Fisher’s exact test and the log-rank test, respectively. Data analyses were conducted using SAS 9.4 software (SAS Institute, Cary, NC, USA).

## Results

### Patient characteristics

Between July 2018 and May 2019, a total of 37 patients with locally advanced or metastatic ESCCs were screened. Five patients did not meet the inclusion criteria, and 2 patients met the exclusion criteria at the screening. Finally, 30 patients were enrolled in the study and received SCT200 treatment as the FAS and safety set (**Supplementary Figure S1**). The median age was 61 years (range: 40–72 years). Of 30 patients, 28 (93.3%) were male, 30 (100%) had ECOG performance status 1, 13 (43.3%) had lung metastases, 7 (23.3%) had liver metastases, 2 (6.7%) had bone metastases, 20 (66.7%) had undergone 1 round of previous therapy, and 10 (33.3%) had at least 2 rounds of previous therapies. The median follow-up duration was 8.2 months (range, 1.2–17.7 months). The median treatment cycles was 9 (range: 3–37 cycles). Patients’ baseline characteristics were presented in **Table 1**.

**Table 1:** Baseline characteristics

Characteristics	Patients (*n* = 30)
Age (years), median (range)	61 (40–72)
Gender, *n* (%)	
Male	28 (93.3)
Female	2 (6.7)
ECOG performance status, *n* (%)	
0	0
1	30 (100)
Location of the primary tumor, *n* (%)	
Upper	6 (20.0)
Middle	14 (46.7)
Lower	9 (30.0)
Middle and lower	1 (3.3)
Site of metastases, *n* (%)	
Brain	0
Liver	7 (23.3)
Lung	13 (43.3)
Bone	2 (6.7)
Others	27 (90.0)
Number of organs with metastases, *n* (%)	
1	11 (36.7)
2	13 (43.3)
≥ 3	6 (20.0)
Prior cancer treatment, *n* (%)	
Surgery	15 (50.0)
Radiotherapy	18 (60.0)
Chemotherapy	30 (100)
Differentiation, *n* (%)	
Well	1 (3.3)
Moderate	13 (43.3)
Poor	6 (20.0)
Unknown	10 (33.3)
Expression of EGFR, *n* (%)	
< 50%	10 (33.3)
≥ 50%	20 (66.7)
Prior therapy lines, *n* (%)	
1	20 (66.7)
2	6 (20.0)
≥ 3	4 (13.3)

ECOG, Eastern Cooperative Oncology Group; EGFR, epidermal growth factor receptor.

### Efficacy

**Figure 1:**
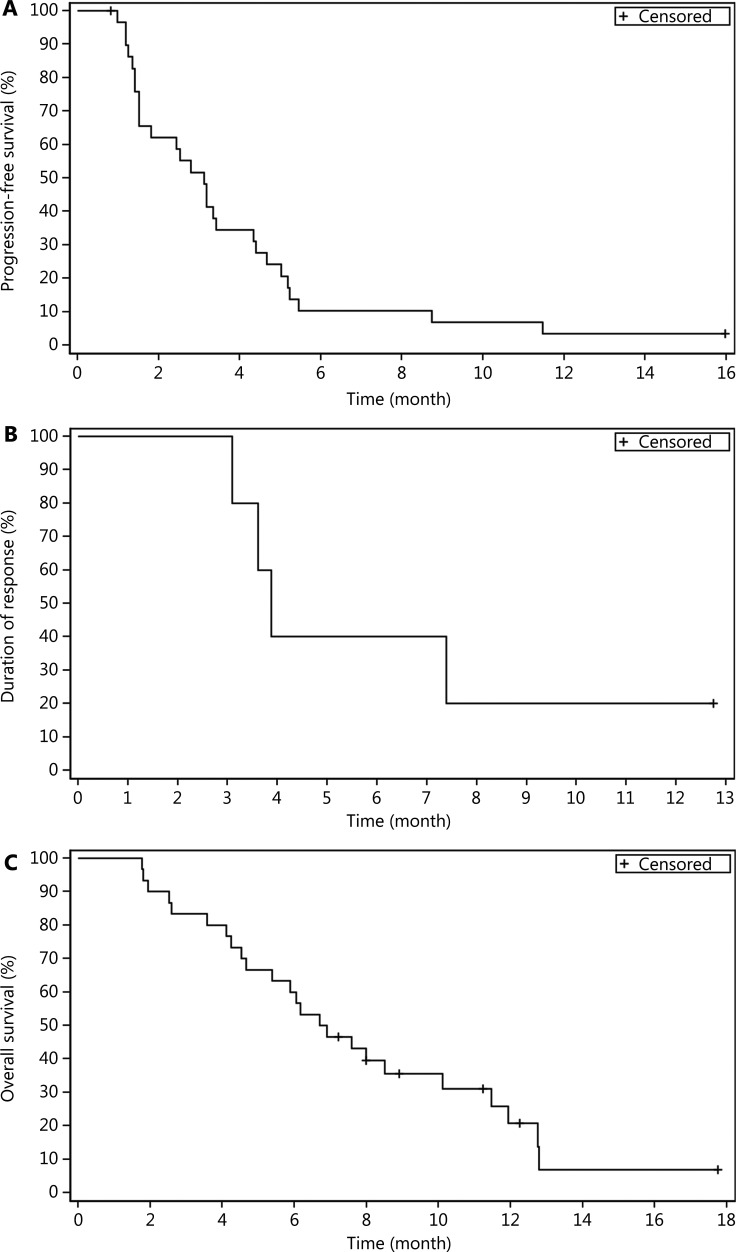
Progression-free survival (A), duration of response (B), and overall survival (C) in the full analysis set.

**Figure 2:**
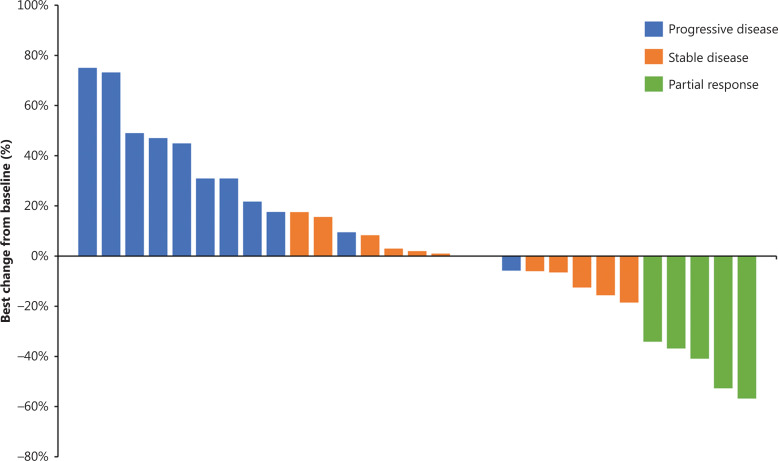
Waterfall plots of best percentage change in targeted lesions for individual patients.

A total of 5 patients achieved PR, with an ORR of 16.7% (95% CI: 5.6%–34.7%). The DCR was 60.0% (95% CI: 40.6%–77.3%) (**Table 2**). The median DOR was 3.9 months (95% CI: 3.1–not reached). The median TTP was 3.2 months (95% CI: 1.5–4.4). Twenty-eight (93.3%) patients had disease progression or died, with a median PFS of 3.1 months (95% CI: 1.5–4.3). Twenty-four (80.0%) deaths occurred, with the median OS of 6.8 months (95% CI: 4.7–10.1) (**Figure 1**). The waterfall plot of best changes in the targeted lesions from the baseline for individual patients is shown in **Figure 2**.

**Table 2:** Efficacy endpoints

	Patients (*n* = 30)
Best tumor response, *n* (%)	
CR	0
PR	5 (16.7)
SD	13 (43.3)
PD	11 (36.7)
NE	1 (3.3)
ORR, *n* (%) [95% CI]	5 (16.7) [5.6–34.7]
DCR, *n* (%) [95% CI]	18 (60.0) [40.6–77.3]
PFS (months), median (95% CI)	3.1 (1.5–4.3)
OS (months), median (95% CI)	6.8 (4.7–10.1)
DOR (months), median (95% CI)	3.9 (3.1–NR)
TTP (months), median (95% CI)	3.2 (1.5–4.4)

CR, complete response; PR, partial response; SD, stable disease; PD, progressive disease; NE, unevaluable; ORR, objective response rate; DCR, disease control rate; PFS, progression-free survival; OS, overall survival; DOR, duration of response; TTP, time to progression.

**Figure 3:**
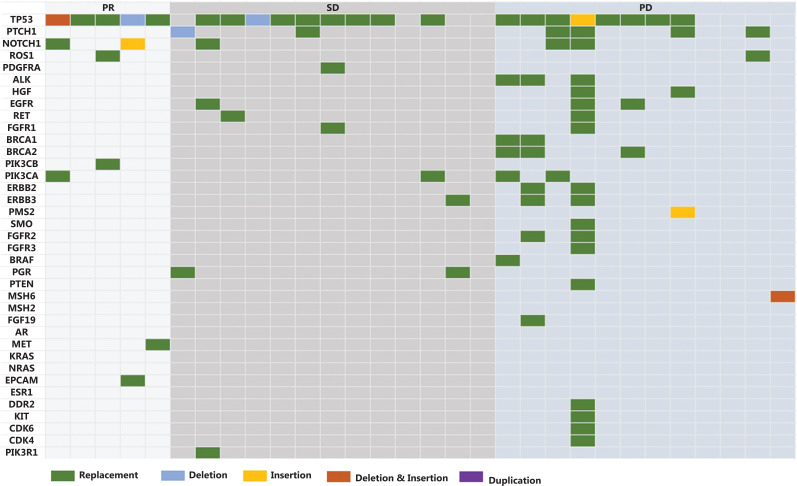
Genomic profiles. PR, partial response; SD, stable disease; PD, progressive disease.

The genomic profiles are shown in **Figure 3**. TP53 mutations were found in 27 (90.0%) patients. Except for 2 (7.4%) patients with extremely low abundances of TP53 mutations and 2 (7.4%) patients with unknown mutation abundances, the changes in the sum of diameter (SOD) of targeted lesions and mutation abundance at different time points revealed similar trends in 21 patients (77.8%, including all 5 patients with PR) and opposite trends in 2 patients (7.4%) (**Supplementary Figure S2**). The occurrence of new lesions explained the opposite trends in the 2 patients with increased mutation abundancies, but decreased SOD of targeted lesions at the time point of PD.

Following subgroup analyses, differences in the ORRs were observed in patients with different EGFR expressions (≥ 50%: 25.0% *vs.* < 50%: 0%, *P* = 0.140) (**Supplementary Figure S3**) or TP53 mutation abundance (< 10%: 23.8% *vs.* ≥ 10%: 0%, *P* = 0.286), but without statistical significance (**Supplementary Table S1**). Patients with high expressions of EGFR showed a tendency of increased PFS (≥ 50%: 3.3 months *vs.* < 50%: 2.5 months, *P* = 0.372) and OS (≥ 50%: 7.4 months *vs.* < 50%: 5.4 months, *P* = 0.306) compared with those with low to moderate expressions of EGFR, but failed to reach a statistical significance (**Supplementary Figure S4**). Longer median PFS (< 10%: 3.4 months *vs.* ≥ 10%: 1.4 months, *P* = 0.006) and OS (< 10%: 8.0 months *vs.* ≥ 10%: 4.2 months, *P* = 0.027) were observed in patients with low abundances of TP53 mutations, when compared with those with high abundances of TP53 mutations (**Supplementary Figure S5**).

### Safety

One patient reported a serious adverse event of esophageal bleeding, which was unrelated to SCT200. The summary of TRAEs is shown in **Table 3**. Any-grade TRAEs were reported in 29 (96.7%) patients, and grade 3 or 4 TRAEs occurred in 10 (33.3%) patients. The most common TRAEs (occurring in ≥ 20% of patients) were hypomagnesemia [*n* = 20 (66.7%)], rash [*n* = 13 (43.3%)], elevated AST [*n* = 9 (30.0%)], increased blood alkaline phosphatase [*n* = 8 (26.7%)], and proteinuria [*n* = 8 (26.7%)]. The most frequently reported grade 3 or 4 TRAEs (occurring in at least 2 patients) were hypomagnesemia [*n* = 7 (23.3%)] and rash [*n* = 2 (6.7%)]. No treatment-related deaths occurred.

**Table 3:** Summary of any-grade TRAEs occurring in ≥ 5% of patients and all TRAEs of grade 3 or 4

	Patients (*n* = 30)	
	Any grade, *n* (%)	Grade 3 or 4, *n* (%)
Any TRAE	29 (96.7)	10 (33.3)
Hypomagnesemia	20 (66.7)	7 (23.3)
Rash	13 (43.3)	2 (6.7)
Increased aspartate transferase	9 (30.0)	0
Increased blood alkaline phosphatase	8 (26.7)	0
Proteinuria	8 (26.7)	0
Acneiform dermatitis	5 (16.7)	1 (3.3)
Increased alanine transferase	5 (16.7)	0
Increased γ-glutamyl transferase	5 (16.7)	0
Infusion-related reaction	4 (13.3)	0
Hypocalcemia	3 (10.0)	1 (3.3)
Increased blood bilirubin	3 (10.0)	0
Decreased neutrophil count	3 (10.0)	0
Paronychia	3 (10.0)	0
Asthenia	2 (6.7)	1 (3.3)
Hypertension	2 (6.7)	1 (3.3)
Acne	2 (6.7)	0
Anemia	2 (6.7)	0
Nausea	2 (6.7)	0
Oral mucositis	2 (6.7)	0
Decreased white blood cell count	2 (6.7)	0
Fatigue	1 (3.3)	1 (3.3)
Lung inflammation	1 (3.3)	1 (3.3)

TRAE, treatment-related adverse event.

### Immunogenicity

The ADAs of all patients in the FAS were negative at baseline. The ADAs were negative for patients with available ADA results after the first 6 week treatment (*n* = 18) and at the end of the treatment (*n* = 25).

## Discussion

SCT200 is a new recombinant all-human, anti-human EGFR developed by Sinocelltech Ltd., Beijing, China. As an anti-human EGFR monoclonal antibody, SCT200 has the same target as cetuximab and panitumumab. However, the antigen-binding epitope, physicochemical properties, and biological activities of SCT200 differ from those of other available anti-EGFR antibodies. SCT200 is a human IgG_1_ monoclonal antibody, which means it has an improved safety profile and higher efficacy than chimeric IgG_1_ monoclonal antibody (cetuximab) and human IgG_2_ monoclonal antibody (panitumumab). Preclinical studies have shown that the antibody has a high affinity (Kd = 0.08 nM), which is higher than cetuximab (0.147 nM) and nituzumab (1 nM), and comparable with that of panimab (0.05 nM). Earlier studies have shown that SCT 200 more effectively inhibited cell growth *in vitro*, and showed a better anti-tumor effect *in vivo*, when compared with cetuximab. In the present study, the safety and efficacy of SCT200 in ESCC patients were determined. In our phase Ib trial, patients with locally advanced or metastatic ESCC who were refractory or intolerant to chemotherapy with platinum, taxane, or fluoropyrimidine were treated with SCT200. The results showed that SCT200, with an ORR of 16.7%, was feasible as a second- or further-line treatment. The median PFS and OS were 3.1 months and 6.8 months, respectively. Moreover, the safety profile was acceptable.

A randomized phase III trial (ESWN 01), with a similar population to that in our study, compared double agent chemotherapy (irinotecan plus S-1) to single agent chemotherapy (S-1 alone) as a second- or further-line treatment in 123 patients with recurrent or metastatic ESCCs^23^. The study reported an ORR of 24.6% with irinotecan plus S-1, 9.7% with S-1 alone, and 17.1% in the whole randomization cohort treated with chemotherapy^23^. SCT200 treatment resulted in a similar ORR to chemotherapy in the ESWN 01 study. Two pivotal randomized phase III trials (ATTRACTION-3 and ESCORT) have been conducted to compare the efficacy and safety of nivolumab or camrelizumab *vs.* our chemotherapeutic drug (paclitaxel or docetaxel in ATTRACTION-3, and docetaxel or irinotecan in ESCORT) in patients with previously treated advanced ESCC^8,24^. In another pivotal randomized phase III trial (KEYNOTE-181) of pembrolizumab *vs.* chemotherapy (paclitaxel, docetaxel, or irinotecan) in patients with previously treated advanced esophageal cancer, subgroup analysis was conducted for ESCC^9^. The ORR was 19.3% with nivolumab^24^, 20.2% with camrelizumab^8^, and 16.7% with pembrolizumab^9^ in the second-line treatment. The ORR in the present study using SCT200 was similar to that of 3 ICIs (16.7%–20.2%)^8,9,24^ and the chemotherapy arm (16.7% *vs.* 21.5%) in the ATTRACTION-3 study^24^. It was higher than that of the chemotherapy arm in the ESCORT study (16.7% *vs.* 6.4%)^8^ and KEYNOTE-181 study (16.7% *vs.* 7.4%)^9^. These indirect comparisons suggested that SCT200 is an alternative treatment for patients who are refractory to chemotherapy and for those who refuse chemotherapy. The tumor response to SCT200 was favorable in patients with advanced ESCC. However, imbalanced baseline characteristics between our study and the previously mentioned studies cannot be ignored, so further RCTs are warranted to confirm the benefits of SCT200, when compared to other therapies. The data of anti-EGFR monoclonal antibody therapy in advanced ESCC patients were limited, although anti-EGFR antibodies have been investigated in patients with ESCC. In a multicenter, phase 2/3 randomized trial (SCOPE1), a treatment regimen of chemoradiotherapy with or without cetuximab was tested in patients with non-metastatic esophagus cancer, which showed that the addition of cetuximab to standard chemotherapy and radiotherapy should not be recommended for patients with esophageal cancer suitable for definitive chemoradiotherapy^25^. In another phase 3 randomized clinical trial, the results showed that the addition of cetuximab to concurrent chemoradiation did not improve overall survival, suggesting minimal benefit to current EGFR-targeted agents in an unselected patient population^18^. However, a few previous studies have reported promising efficacies of anti-EGFR monoclonal antibodies (such as cetuximab and nimotuzumab) plus chemoradiotherapy in advanced ESCC patients using single-arm trials, with an ORR of 51.8%–100%^11,14–17^, which indicated the benefits of combination therapy. In a phase 2 study evaluating the long-term outcome of nimotuzumab plus paclitaxel and cisplatin as a first-line treatment in patients with esophageal cancer, the results also showed that a combination of nimotuzumab plus paclitaxel and cisplatin was effective as a first-line treatment for patients with unresectable and metastatic ESCCs^26^. However, there is an unmet requirement of chemotherapy-free therapy for patients with ESCC who cannot tolerate chemotherapy or do not respond to immunotherapy. As a single-agent therapy, a multicenter phase II trial of cetuximab in advanced ESCC patients showed that cetuximab administered as a single agent provided minimal clinical benefit in patients with metastatic esophageal adenocarcinomas^27^. The clinical activity of cetuximab as second-line therapy in patients with metastatic esophageal adenocarcinoma has also been evaluated, which showed that cetuximab alone should not be recommended for second-line treatment of metastatic esophageal cancer due to failure in improving the clinical outcomes^28^. In contrast, the results in our study showed that SCT200 had great advantages when compared to cetuximab, and could provide more clinical benefits to patients with advanced ESCCs. The results highlighted that SCT200 is a promising treatment agent either alone or in combination with other therapeutic agents. Additionally, it is reasonable to assume that patients with advanced ESCC or patients refractory to conventional chemotherapy could benefit from SCT200 treatment.

The median OS was 7.1 months with irinotecan plus S-1 and 6.2 months with S-1 alone in the ESWN 01 study^23^. The ATTRACTION-3, ESCORT, and KEYNOTE-181 studies had median OSs of 8.4, 6.2, and 7.1 months with our selected chemotherapeutics, respectively, and 8.2–10.9 months with ICIs, respectively^8,9,24^. Randomized phase III trials showed that the median OSs with nivolumab, camrelizumab, and pembrolizumab in patients with previously treated advanced ESCC were 10.9, 8.3, and 8.2 months^8,9,24^, respectively. The median OS with chemotherapy in those trials was 8.4, 6.2, and 7.1 months^8,9,24^, respectively. Although the survival benefits of SCT200 were not comparable to those of immunotherapy, the median OS of 6.8 months was comparable to that of chemotherapy. In contrast, the success of combination therapy with anti-EGFR monoclonal antibody (cetuximab or nimotuzumab) plus chemoradiotherapy in single-arm trials^11,14–17^, but their failure in RCTs^18,19^, has provided us certain treatment guidelines. The phase II NICE study involving 93.5% of patients with locally advanced ESCC did not result in survival benefits when nimotuzumab was added to chemoradiotherapy^19^. This could be attributed to inadequate sample size for comparing the OS between groups, because it was calculated based on the primary endpoint (endoscopic complete response percentage) rather than the OS. Nevertheless, a tendency of increased survival benefit with a numerically longer median OS (15.9 *vs.* 11.5 months) was observed in the group with nimotuzumab plus chemoradiotherapy, when compared with chemoradiotherapy alone^19^. In the phase III NRG Oncology RTOG 0436 study, OS was set as the primary endpoint; however, it failed to show a survival benefit with the addition of cetuximab to chemoradiotherapy^18^. The enrollment of 19.8% of patients with T1/2 disease and 61.9% with an adenocarcinoma subtype resulted in good prognoses for both groups, which diluted the effect of cetuximab, and resulted in no difference in the median OS between the groups (19.7 months in the cetuximab plus chemoradiotherapy group and 19.0 months in the chemoradiotherapy group)^18^. These failures showed us that we should target selected patients with ESCC when using anti-EGFR monoclonal antibodies. Moreover, predictive biomarkers are also required.

Previous studies showed that high expression of EGFR was associated with poor prognoses in ESCC patients^11–13^. We analyzed the effect of EGFR expression on ORR and survival. Numerical differences in ORR (25.0% *vs.* 0%) and OS (7.4 *vs.* 5.4 months) were observed between patients with high expressions of EGFR and those with low to moderate expressions of EGFR. Because the small sample size affected the statistical difference significance, the predictive function of EGFR expression in patients with ESCC treated with SCT200 still requires validation in large-scale studies. The results from another subgroup analysis revealed that a low abundance of TP53 mutation was associated with improved PFS and OS. Moreover, we found a potential correlation between *TP53* mutation abundance and SOD of targeted lesions. These findings are supported by those of previous studies showing that *TP53* gene mutations were associated with esophageal squamous cell carcinogenesis and resulted in more aggressive diseases and poor prognoses^29,30^. Furthermore, the use of biomarkers, such as *TP53* mutation abundance, could guide targeted therapy of ESCC patients.

The TRAE profile of SCT200 was similar to that of other anti-EGFR monoclonal antibody therapies, such as cetuximab^31,32^, nimotuzumab^33^, and panitumumab^34–36^, for solid cancers. All TRAEs were predictable and manageable. No treatment-related death was reported, and no unexpected safety signal was identified.

The present study had certain limitations. First, this was a single-arm trial without a control group. Bias could not be avoided using historical data as controls. Second, the sample size was relatively small. However, based on the promising results, we recommend that further large-scale clinical trials be conducted.

## Conclusions

SCT200 monotherapy as second- or further-line treatment for advanced ESCC showed favorable efficacies, with an acceptable safety profile. This treatment could be an alternative option for patients with ESCC who showed no response to immunotherapy or could not tolerate chemotherapy, warranting further studies. In addition, predictive biomarkers, such as *TP53* mutation abundance, should be further studied.
