# Supplementary material for: Safety and efficacy of anti-EGFR monoclonal antibody (SCT200) as second-line therapy in advanced esophageal squamous cell carcinoma
Source: Cancer Biol Med. 2022 Jan 12;19(3):358–69. doi: 10.20892/j.issn.2095-3941.2021.0388 (PMC8958882; doi:10.20892/j.issn.2095-3941.2021.0388)
Supplement: Supplementary file 1 [file cbm-19-358-s001.pdf]

Supplementary materials

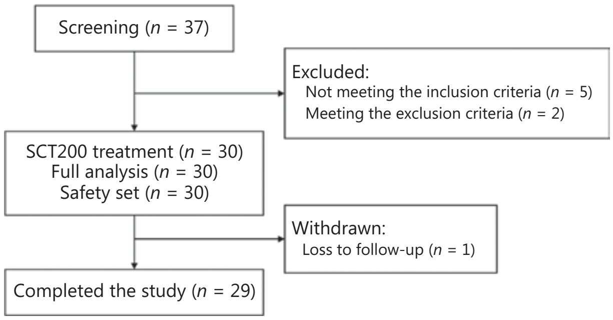

Figure S1 Flowchart of the work.

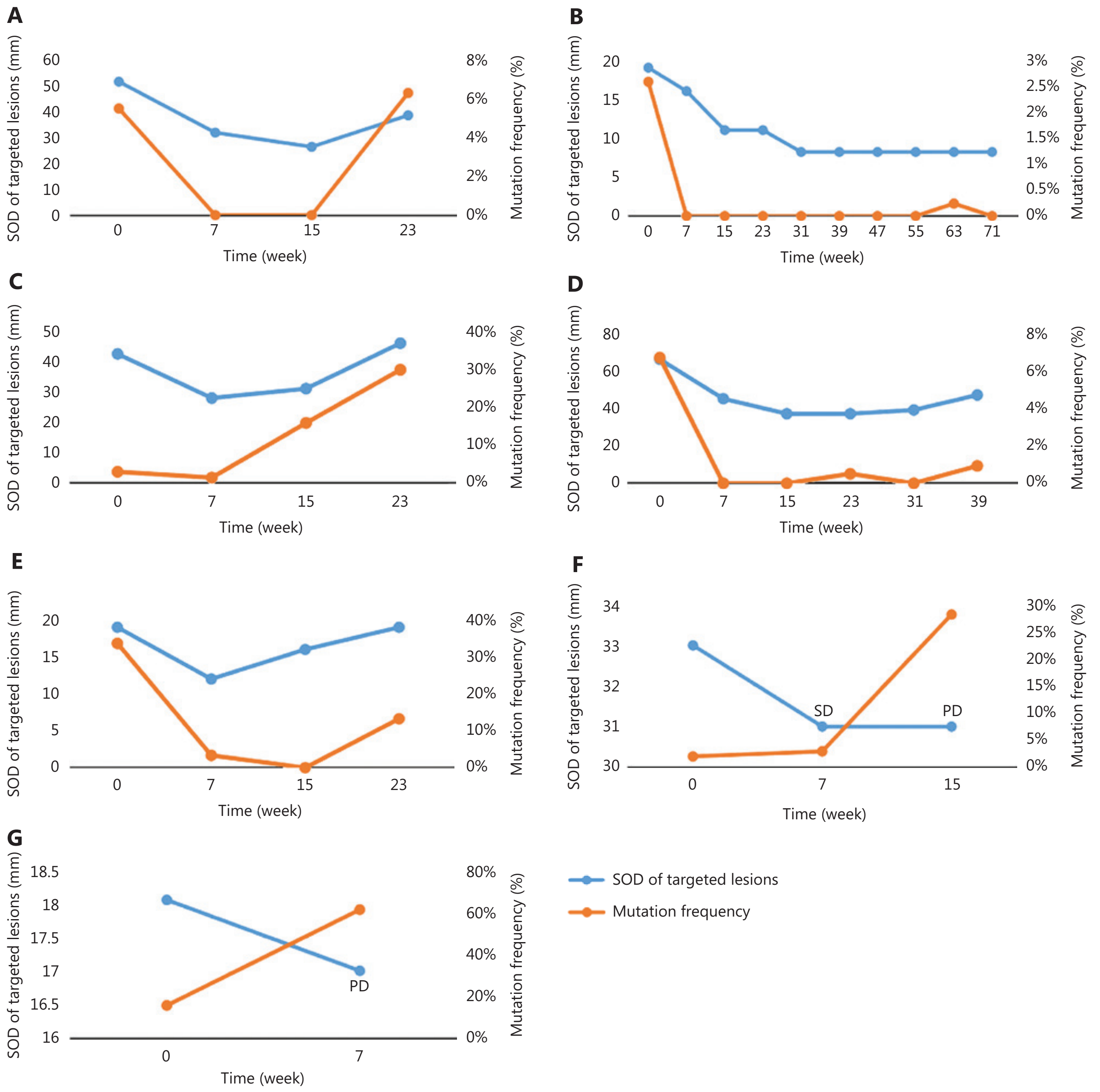

Figure S2 Changes in the SOD of targeted lesions and mutation abundance at different time points in five patients with a partial response showing similar trends (A-E) and two patients with PD showing opposite trends (E-F). SOD, sum of diameter; SD, stable disease; PD, progressive disease.

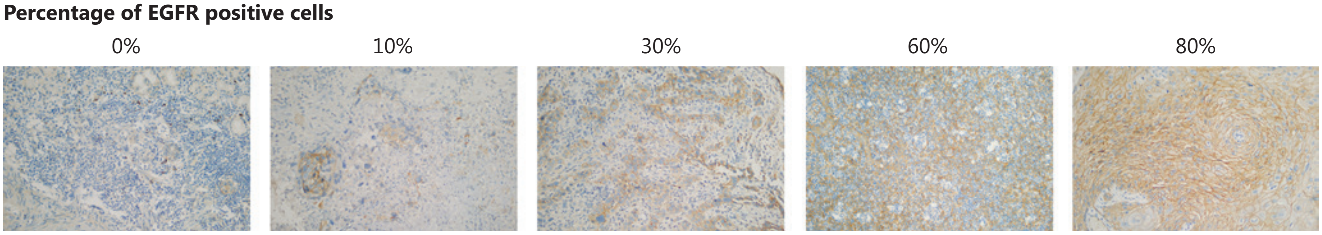

Figure S3 The representative images of immunochemical staining of EGFR in ESCC specimens. The number displayed were percentage of EGFR positive stained cells of each specimen.

Table S1 ORR by EGFR expression and *TP53* mutation abundance

	PR	SD	PD	NE	ORR	*P*-value
EGFR expression, *n* (%)						0.140
< 50% (*n* = 10)	0	5 (50.0)	4 (40.0)	1 (10.0)	0	
≥ 50% (*n* = 20)	5 (25.0)	8 (40.0)	7 (35.0)	0	5 (25.0)	
TP53 mutation abundance, *n* (%)						0.286
< 10% (*n* = 21)	5 (23.8)	11 (52.3)	5 (23.8)		5 (23.8)	
≥ 10% (*n* = 9)	0	2 (22.2)	6 (66.7)	1 (11.1)	0	

EGFR, epidermal growth factor receptor; PR, partial response; SD, stable disease; PD, progressive disease; NE, unevaluable; ORR, objective response rate.

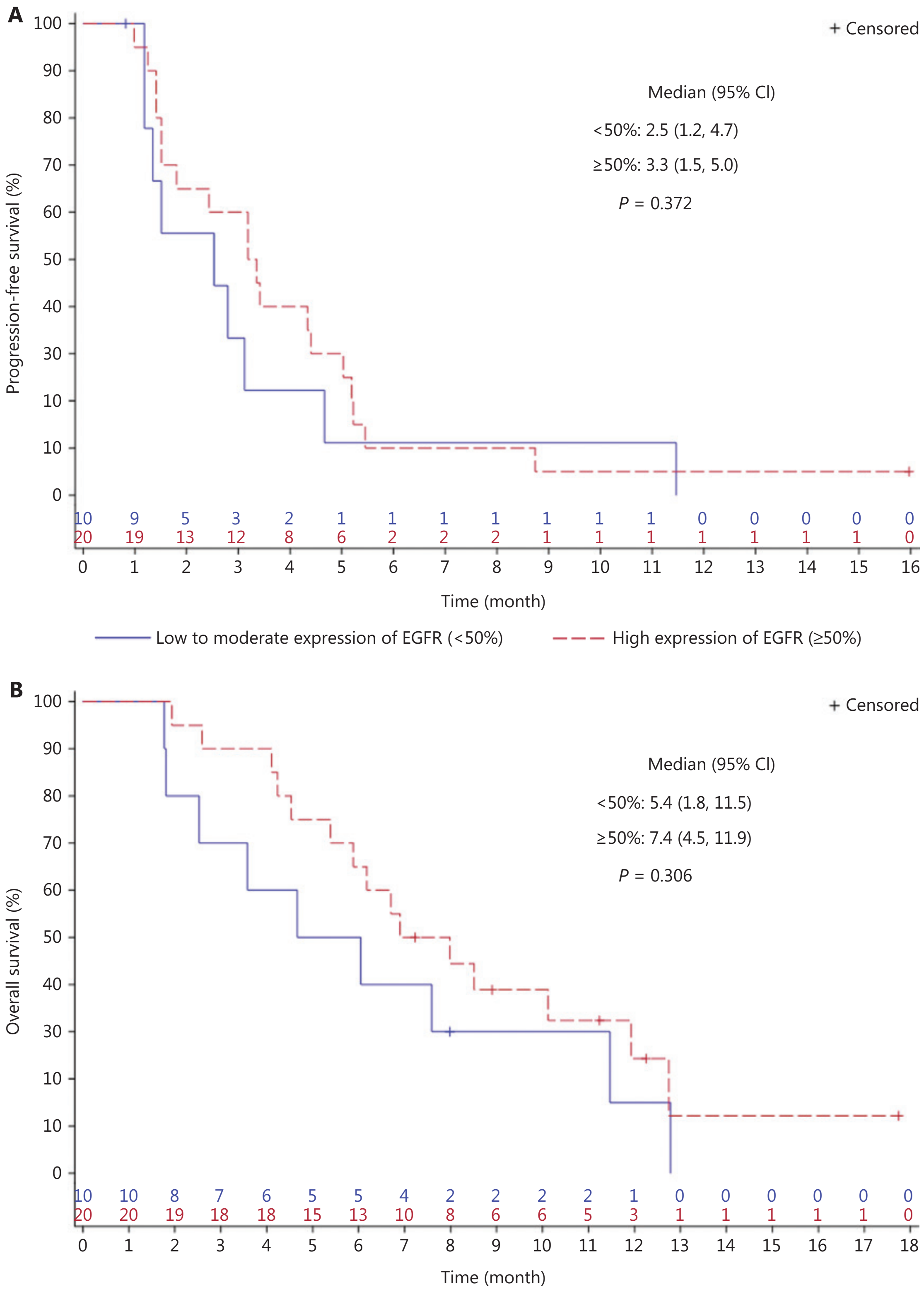

Figure S4 Progression-free survival (A) and overall survival (B) in patients with different EGFR expressions. EGFR, epidermal growth factor receptor.

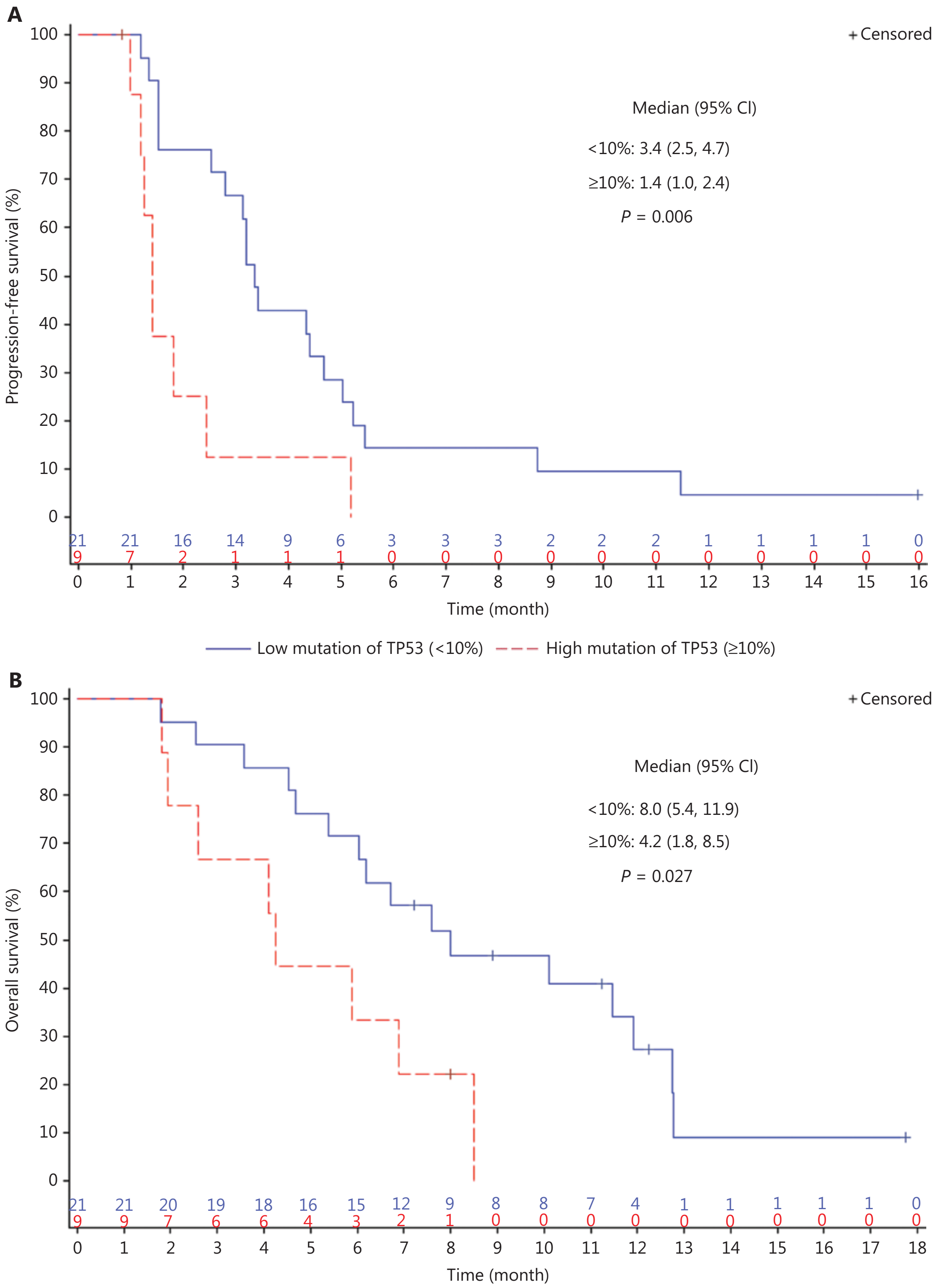

Figure S5 Progression-free survival (A) and overall survival (B) in patients with different TP53 mutation abundance.
